# Cross-sectional survey to inform the development of a telehealth support model: a feasibility study for women undergoing breast cancer surgery

**DOI:** 10.1186/s40814-019-0426-5

**Published:** 2019-03-16

**Authors:** Natasha Noble, Lisa Mackenzie, Mariko Carey, Anthony Proietto, Robert Sanson-Fisher, Gail Walker, Judith Silcock

**Affiliations:** 1 School of Medicine and Public Health, Faculty of Health and Medicine, University of Newcastle, Callaghan, NSW 2308 Australia; 2 Priority Research Centre for Health Behaviour, University of Newcastle, Callaghan, NSW 2308 Australia; 3 Hunter Medical Research Institute, New Lambton Heights, NSW Australia; 4 Hunter New England Local Health District, New Lambton Heights, NSW Australia; 5 Breast and Endocrine Centre, Gateshead, NSW Australia

**Keywords:** Breast cancer, Surgery, Information needs, Preparation, Telehealth, Feasibility, Psychosocial

## Abstract

**Background:**

For patients undergoing breast cancer surgery, the pre- and post-operative periods can be characterised by feelings of fear, anxiety, and uncertainty. Telehealth offers an opportunity to provide perioperative support to surgical patients and overcome some of the barriers to accessing care.

**Aims:**

In order to inform the development of a telehealth support model for women undergoing breast cancer surgery, this feasibility study explored: (a) access and preferences for telehealth; and (b) the proportion of participants who reported problems with unmet information and preparation needs related to surgery, post-operative pain, anxiety, and quality of life.

**Methods:**

Women aged 18–85 years attending for a follow-up appointment within 2 months of undergoing surgery for breast cancer were asked to complete a baseline (T1) and 1-month follow-up (T2) survey. Surveys assessed telehealth access and preferences, preparatory information receipt and preparedness for surgery, and anxiety, pain, and quality of life.

**Results:**

Fifty-three T1 (45% consent rate) and 50 T2 surveys were returned. One fifth of the sample (20%) travelled 50 km or more to access surgery. Most participants had access to a device suitable for telehealth (75%); however, only 15% indicated that they would have accepted a teleconsultation with their surgeon post-operatively if this had been offered. The most frequently reported unmet preparatory information needs were information about: how long it would take to recover from the surgery; how other patients had experienced similar surgery; and practical needs such as parking or transport. Approximately one third of the sample reported potentially clinically significant symptoms of anxiety, and less than one in ten women reported moderate levels of pain.

**Conclusions:**

While the majority of women had access to a suitable device and internet connection for telehealth, less than one fifth would have accepted a home-based video-link teleconsultation with their surgeon post-operatively. A small proportion of the sample would have liked more information about aspects of surgery including about managing side effects and anxiety. The key findings in terms of teleconsultation preferences and information and preparation needs from this study will be incorporated into the telehealth support model being developed.

## Background

In 2013, breast cancer was the second most commonly diagnosed cancer in Australia and is estimated to become the most commonly diagnosed cancer in 2017 [1]. The majority of patients diagnosed with breast cancer will undergo surgery [2]. For patients undergoing surgery for their breast cancer, the pre- and post-operative periods can be characterised by feelings of fear, anxiety, isolation, and uncertainty [3–5]. The provision of information to guide expectations for surgical procedures and outcomes can improve patient outcomes, including reducing rates of post-operative complications and levels of anxiety and pain [6].

Telehealth includes a variety of technologies and methods to deliver virtual medical, health, and education services, with the aim of enhancing health care [7]. Telehealth presents an opportunity to provide information and support to surgical breast cancer patients [6] and can address a range of psychosocial [8], financial, and practical burdens [9]. For example, web-based information can be accessed when the patient wants and at their own pace [6] and can be presented in multiple formats such as text, images, and videos [10]. Technologies including video-conferencing can allow patients to access consultations at home, reducing the burden and cost associated with travel [9, 11]. Yet, telehealth support has not been widely tested for breast cancer surgery patients in the Australian context. Telehealth may be particularly suitable in this setting, given that almost a third of the population live outside of major cities [12], which often entails travelling large distances to receive breast cancer treatment [13].

This study aimed to explore the acceptability and feasibility of a telehealth model of support for breast cancer surgery patients and assess any issues with unmet information and preparation needs related to surgery, post-operative pain, anxiety, and quality of life, among this population. Results of the study will be used to inform the content and delivery of a telehealth support model including access to an interactive website and to a home-based teleconsultation with participating surgeons using secure video-conferencing software. A randomised controlled trial (RCT) will be undertaken to assess the effectiveness of telehealth support in reducing post-operative levels of anxiety and improving quality of life for women undergoing surgery for breast cancer. Acceptability and feasibility of the telehealth support model was based on the following criteria: 75% of respondents indicating access to equipment suitable for a teleconsultation (including internet access and an internet connected device with a camera and speakers), 50% indicating willingness to accept a post-operative teleconsult, and 50% of respondents indicating at least one unmet informational or preparation need related to surgery. These criteria were based on sample size considerations for the planned RCT.

### Aims

To explore the acceptability and feasibility of a telehealth model of support among a sample of women who had recently undergone surgery for breast cancer. In order to inform the content of the planned telehealth model, we also explored the proportion of participants who reported problems with unmet information and preparation needs related to surgery and post-operative pain, anxiety, and quality of life.

## Methods

### Study design and setting

A cross-sectional survey was conducted within a privately run breast cancer clinic located in a major city in New South Wales (NSW). The clinic sees approximately 2000 new and follow-up breast cancer patients per year. Data were collected in 2016–2017. Ethical approval for the study was obtained from the University of Newcastle Human Research Ethics Committee (Ref: H-2015-0412) and all participants provided written informed consent.

### Participants and procedure

Women aged 18–85 years, attending for a follow-up appointment within 2 months of undergoing surgery for breast cancer, able to complete a survey in English, and considered mentally and physically well enough by their Breast Care Nurse, were asked to complete a baseline (T1) and 1-month follow-up (T2) survey. Women were informed about the study by their nurse when they attended the clinic for a post-surgery appointment and were provided with a study information package to take home. The information package contained a study information statement, consent form, copy of the T1 survey, and a reply paid envelope. Consenting participants were asked to complete the consent form and survey and return these to the researchers using the envelope supplied. Participants who completed the consent form were sent the second (T2) survey and a reply paid envelope 1 month later. Participants received up to two phone call reminders if they had not returned the second survey within 2 weeks.

### Measures

#### Demographics and treatment variables (T1 survey)

Participants were asked to self-report their age, marital, employment and Aboriginal or Torres Strait Islander status, highest level of education, whether they had private health insurance or a concession card, type of surgery received, whether they had undergone chemotherapy, and distance travelled for surgery.

#### Acceptability/feasibility of telehealth support (T1–T2 survey)

A subset of participants was asked a series of questions relevant to the provision of a telehealth support model. These included whether they had access to the internet and a device suitable for a teleconsultation (i.e. an internet connected device with a camera and speakers; T1 survey). Participants were given a brief description of a teleconsultation (“In a telehealth appointment, you use a computer, tablet or smart phone to have a video-link appointment with your surgeon. You and your surgeon can see and hear each other. It means that you can stay at home rather than having to travel for your appointment”) and asked whether or not they would have accepted a teleconsultation follow-up appointment immediately after their surgery, instead of an in-person appointment, if their surgeon had offered this to them (T2 survey). Response options were ‘yes’, ‘no’ and ‘not sure’.

#### Preparedness for surgery (T1)

**Fig. 1:**
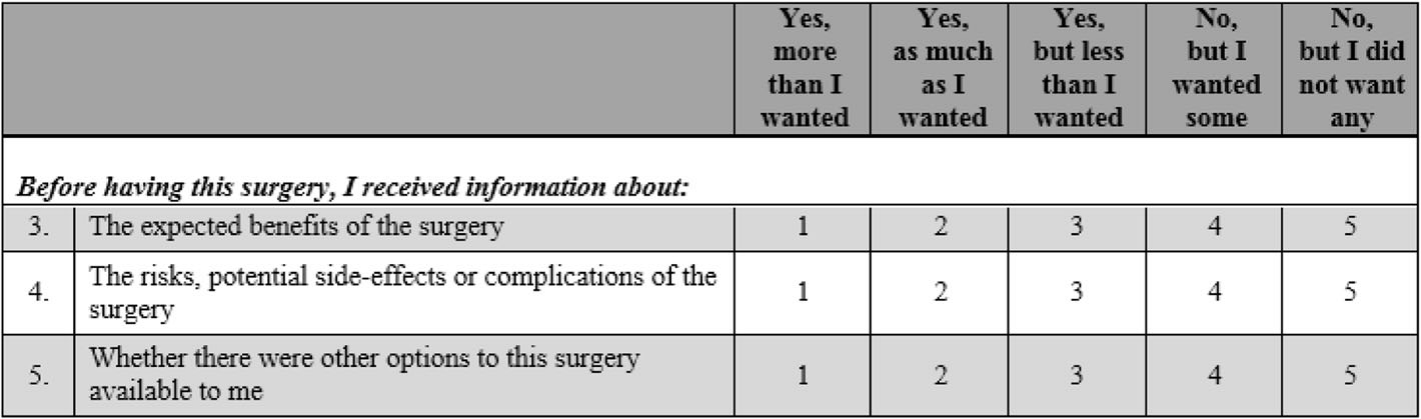
Example items from module 1 of the MiPrep tool (information receipt)

‘The MiPrep tool’ is a 27-item measure with two modules, developed to measure patients’ perceptions about their preparation for medical interventions. The first module (18 items) asked patients whether they received as much information as they would have liked on a range of preparatory aspects of surgery. Some examples of the types of items and the response options are shown below in Fig. 1. The response options 3 (‘Yes, but less than I wanted’) and 4 (‘No, but I wanted some’) were used to indicate a ‘gap’ or unmet need in information provision.

**Fig. 2:**
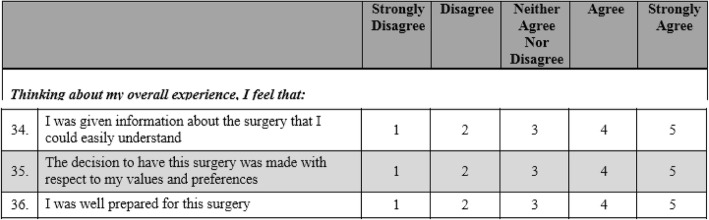
Example items from module 2 of the MiPrep tool (overall preparation for surgery)

The second module of the MiPrep tool (nine items) examined patient’s overall appraisal of their preparation for surgery, with participants asked about their level of agreement with statements such as “I was well prepared for my surgery”. An example of some of the items and response options are shown in Fig. 2. The response options 1 (‘strongly disagree’) and 2 (‘disagree’) were used to indicate inadequate preparation for surgery.

The MiPrep tool has recently been validated in an Australian sample of patients undergoing radiotherapy or a medical imaging intervention (such as an MRI and CT scan), of which a significant proportion had a suspected or confirmed diagnosis of cancer (55%) [14]. The tool demonstrated face and content validity, good internal consistency, and acceptable test-retest reliability [14].

#### Pain (T1)

Pain was assessed using the visual analogue scale [15], a horizontal line ranging from 0 (no pain), to 5 (moderate pain), to 10 (worst possible pain). Participants were asked to indicate their level of pain in the past 24 hours by placing a mark on the line. The VAS has been used extensively in the assessment of pain intensity, including for cancer patients and in the assessment of postoperative pain [16]. Paul et al. [17] recommended the following optimal cut-off scores for cancer patients: mild pain, 1–4; moderate pain, > 4–7; and severe pain, > 7–10. Cut-off scores are based on patient ratings of pain interference with functions such as general activity, mood, and sleep.

#### Anxiety (T1 and T2 surveys)

Anxiety was measured using the State subscale of the State-Trait Anxiety Inventory (STAI) Form Y [19]. The STAI State subscale (STAI-S) includes 20 items assessing current levels of anxiety or worry [19]. Higher scores on the STAI-S indicate higher levels of anxiety. There is extensive data supporting the reliability and validity of the STAI [20], and it has been widely used in clinical and medical populations including for assessment of preoperative anxiety among breast cancer patients [21] and post-operative anxiety among cancer outpatients [20]. In the current study, anxiety was measured at two time points (baseline and follow-up) in order to explore any changes in levels of anxiety over the post-operative period. A cut-point of 39–40 has been suggested to detect clinically significant symptoms for the STAI anxiety scale [22].

#### Quality of life (T2)

Quality of life was measured using the 37-item Functional Assessment of Cancer Therapy-Breast (FACT-B) tool, which assesses health-related quality of life across multiple subscales including physical, social, emotional, and functional wellbeing, and ten items specific to breast cancer concerns [23]. Scores on the FACT-B can range from 0 to 160. Higher scores on the FACT-B indicate better quality of life. The FACT instrument has excellent reliability and validity [24].

### Analysis

Means and standard deviations were calculated for continuous variables and proportions with confidence intervals for categorical variables. Imputation using the within-participant mean response to prorate the total score was used for missing values on the STAI-S, where < 10% of responses was missing. Scores were also prorated using subscale mean scores on the FACT-B where < 50% of subscale responses was missing. Participants were excluded from analysis if more than 10% or more than 50% of subscale responses were missing for the STAI, or the FACT-B and MiPrep tool, respectively. A sample size of 50 allowed the outcomes to be calculated with a 95% confidence level and 10% precision, assuming a prevalence rate of 50% reporting at least one unmet information or preparation need on the MiPrep tool.

## Results

A total of 117 patients were given a study information package, with 53 participants returning a baseline (T1) survey (45% consent rate) and 50 returning a follow-up (T2) survey (94% retention rate). The length of time between T1 and T2 survey completion was approximately 6 weeks. The main characteristics of the sample are shown in Table 1. The majority of the sample were aged 60 years and over and had undergone breast-conserving surgery. The vast majority of the sample had private health insurance. One in five participants travelled more than 50 km for treatment.

**Table 1:** Demographics characteristics of the baseline study sample (*n*?=?53)

Demographic characteristic	Sample *n* (%)^a^
Age	
40–49 years	5 (9%)
50–59 years	12 (23%)
60–69 years	20 (38%)
70 years and over	16 (30%)
Aboriginal or Torres Strait Islander status	
Aboriginal or Torres Strait Islander	–
Neither Aboriginal or Torres Strait Islander	52 (100%)
Highest level of education	
High school (year 12 or below)	17 (32%)
University	18 (34%)
Other	18 (34%)
Employment status	
Employed (full or part time)	16 (30%)
Retired	30 (57%)
Other	7 (13%)
Private health insurance	
Yes	48 (91%)
No	4 (9%)
Concession card	
Yes	27 (51%)
No	26 (49%)
Type of surgery	
Breast-conserving surgery (lumpectomy)	33 (62%)
Mastectomy	20 (38%)
Type of node surgery	
Sentinel node biopsy	24 (45%)
Axillary node dissection	6 (11%)
None	23 (43%)
Chemotherapy	
Yes, or planning to	13 (24%)
No	40 (76%)
Distance travelled for surgery	
<?20 km	28 (53%)
20–49 km	14 (27%)
50–100 km	6 (12%)
>?100 km	4 (8%)

^a^Totals may not add to the total sample due to missing values

### Acceptability and feasibility of telehealth support (*n* = 20)

Almost all of the subsample of participants asked about telehealth variables had either broadband (79%) or other internet (11%) access at home. Three quarters (75%) of respondents had a device suitable for telehealth (i.e. an internet connected device with a camera and speakers). Only 15% of the subsample indicated that they would have accepted a teleconsultation, 20% were not sure, and 65% indicated they would not have accepted a teleconsult with their surgeon instead of an in-person follow-up consultation immediately following their surgery, if this had been offered.

### Proportion of the sample reporting problems with unmet information and preparation for surgery needs, post-operative pain, anxiety, and quality of life

#### Unmet information and preparation for surgery needs (T1, *n* = 53)

A total of 5 participant scores on the MiPrep tool were prorated, and no participants were excluded. Just over half of the sample (53%) reported no unmet information or preparation needs, with the remaining sample reporting at least one unmet need. Table 2 shows items where more than 10% of the sample indicated they would have liked more information than they received (module 1) on the MiPrep tool. Table 3 shows items where more than 10% of the sample reported feeling inadequately prepared (module 2).

**Table 2:** Module 1—aspects of surgery that women would have liked more information about (reported by =?10% of the sample)

Informational about	Number (%) wanting more information than they received [95% CI]
How other patients had experienced similar surgery	11 (21%) [9–32%]
How long it would take to recover from the surgery	11 (21%) [9–32%]
Strategies to help me manage any anxiety or stress before or during the surgery (e.g. listening to music etc.)	8 (15%) [5–25%]
How to manage any side effects (e.g. fatigue, swelling, or pain) if they occur	8 (15%) [5–25%]
What needs to happen before the surgery (e.g. skin markings, special diet, and anaesthesia)	6 (11%) [3–20%]

**Table 3:** Module 2—general aspects of surgery that women did not feel well prepared for (reported by =?10% of the sample)

Overall preparedness for surgery	Number (%) responding disagree/strongly disagree [95% CI]
My health care providers provided me with information about practical issues (e.g. parking or transport available to me)	10 (19%) [8–29%]
My health care providers explained to me that I could choose whether or not to have the surgery	7 (13%) [4–22%]
My health care providers asked me whether I wanted to have a support person (e.g. family, carer, or close friend) with me	6 (11%) [3–20%]

#### Post-operative levels of pain (T1), anxiety (T1 and T2), and quality of life (T2)

The mean score for pain (T1 only, *n* = 52) on the VAS was 2.1 (SD = 1.6, range 0–6, 1 missing value). Nine participants (17%) reported no pain (scores of 0), 47 participants (90%) reported mild pain (scores of 1–4), and 5 participants (10%) reported moderate levels of pain (scores of > 4–7) [17]. No participants reported severe pain using the cut-off score of > 7 [17]. The mean anxiety score on the STAI-S was 39.9 (SD = 11.6) at T1. Scores for 7 participants were prorated and 2 participants were excluded due to missing values at T1. The mean anxiety score was 35.7 (SD = 11.9) at T2, with 8 scores prorated and 1 participant excluded due to missing values at T2. Using a cut-off score of 39–40 [22], 37% of the sample (*n* = 19/51) had possible clinically significant symptoms of anxiety at baseline (T1) and 32% at follow-up (*n* = 16/50; T2). A paired *t* test showed that mean anxiety scores did not differ significantly between baseline and follow-up (*t* = 0.09, df = 46, *p* = 0.9). Quality of life scores (FACT-B; T2 only) ranged from 58 to 146, with a mean score of 114/160 (SD = 20.9). FACT-B scores for 9 participants were prorated, and no participants were excluded.

## Discussion

This study explored the acceptability and feasibility of telehealth support for a sample of women who had recently undergone surgery for breast cancer. Three quarters (75%) of the sample reported access to suitable resources for telehealth (including internet access and a smart device with camera and speakers). However, only 15% of the sample indicated that they would have accepted a post-operative teleconsultation with their surgeon if this had been offered, with 65% indicating they would not have accepted. This may be due to a lack of familiarity or confidence with telehealth technology [25], or a perception that a teleconsultation would provide poorer quality of care than a face-to-face consult [26]. It is also possible that women would be more willing to accept a teleconsultation offered to them by their surgeon (along with reassurance that it is clinically appropriate for them), compared to being asked the hypothetical question in this feasibility study. Teleconsults offer significant potential advantages including lower costs, improved access to services, and improved quality of clinical services [27], yet our feasibility study findings suggest that offering a teleconsultation for breast cancer surgery patients immediately following surgery is unlikely to be an acceptable alternative to seeing the surgeon in person. It would be useful to explore patient barriers to uptake in future research. Teleconsults may be more acceptable at other times in the care trajectory, such as for a subsequent follow-up consultation. However, further research would also be needed to confirm this.

Just under half of the sample (47%) reported one or more unmet information and preparation needs, similar to the findings of the original MiPrep validation study [14]. The main gaps were related to recovery times, what happens prior to surgery, and how to manage anxiety and the possible side effects of surgery. Women also reported wanting more information about how other patients had experienced similar surgery. Almost one in five women reported moderate levels of pain and approximately one third reported potentially clinically significant levels of anxiety. Anxiety scores were similar to those reported in other studies of women prior to undergoing breast cancer surgery [21, 28]. Quality of life scores were also similar to those reported in studies of breast cancer patients in Australia [24] and the USA [29].

The unmet informational and preparation needs, and the prevalence of pain and anxiety reported in this study, substantiate the need for additional support to be offered to women undergoing surgery for breast cancer. Given the low acceptability of immediate post-operative telehealth consultations in the current study, it may be worth exploring how telehealth strategies could be used to supplement face to face clinical consultations rather than replace them. Due to existing time pressures on healthcare staff, web-based approaches offer significant opportunities to address gaps in information provision [10]. For example, a website can provide information about how to prepare for surgery, estimated recovery times, the potential side effects of surgery, and how these might be managed. Web-based approaches can also be used to offer post-operative support, such as management of side effects and links to available services and resources [10]. To address the specific gaps identified in this study, a website for women undergoing surgery for breast cancer could present videos, written stories, and/or photographs to illustrate patients talking about their own experiences of breast cancer surgery. Guided visual or audio relaxation exercises could also be embedded to help patients manage anxiety related to surgery. Web-based approaches allow tailoring or modification of content [10], so that information that is specific to a person’s circumstances or type of surgery (e.g. lumpectomy versus mastectomy) can be presented. Telehealth approaches, including a teleconsult and website access, could be utilised to address unmet needs and provide additional information as a supplement to, rather than replacement for, existing clinical consultations.

### Limitations

As noted above, this small study sample was drawn from one, privately run treatment centre, located in a major city. The majority of women (53%) did not need to travel more than 20 km for their treatment, and almost all had private health insurance (91%). Therefore, the findings of the study may not be generalisable to the broader population of breast cancer patients in Australia, in particular those located in regional and rural areas [30], and those who access care through the public healthcare system [18], who may be more willing than this sample to accept a telehealth consultation. In addition, the consent rate for the baseline survey was modest, and only a subsample was asked about the acceptability of a telehealth consultation with their surgeon.

## Conclusion

The pilot study demonstrated feasibility but low acceptability of a telehealth support model in relation to uptake of a post-operative teleconsultation. The support model being developed by the study authors will incorporate the telehealth preferences and key unmet information and preparation needs identified in this feasibility study, including online provision of information about recovery times, management of surgery side effects, experiences of other patients who have undergone breast cancer surgery, and tools to help patients manage anxiety related to surgery, as well as the possible supplementation of clinical consults with teleconsultations, and/or flexibility in the timing of these. Testing the support model with a larger and more diverse sample of breast cancer surgery patients using a RCT design will allow us to rigorously explore the effectiveness of telehealth support for improving post-operative levels anxiety and quality of life.
